# Facile Spin-Coated MoS_2_ Thin Films from a Single-Source Precursor for HER Activity

**DOI:** 10.1021/acsaem.5c00619

**Published:** 2025-06-27

**Authors:** Talha Nisar, Muhammad Adeel Asghar, Abu Nasar Siddique, Ali Haider, Kaline Pagnan Furlan, Veit Wagner

**Affiliations:** † School of Science, 84498 Constructor University, Campus Ring 1, 28759 Bremen, Germany; ‡ Hamburg University of Technology (TUHH), Integrated Ceramic-based Materials Systems Group, 21073 Hamburg, Germany; § Department of Chemistry, 66757 Quaid-i-Azam University, Islamabad 45320, Pakistan; ∥ Institute of Biotechnology and Microbiology, 389328 Bacha Khan University Charsadda, Charsadda 24420, Pakistan

**Keywords:** MoS_2_, hydrogen evolution reaction (HER), solution-based deposition, single source precursor, 2D-material, transition metal dichalcogenide (TMD), electrocatalyst

## Abstract

Hydrogen evolution reaction (HER) is one of the most promising ways to replace the consumption of fossil fuels with a clean and green energy source. HER requires a suitable material as a catalyst to lower overpotential and minimize energy consumption. MoS_2_ is an excellent candidate for the HER because of its suitable band structure. It is an economical and earth-abundant material compared to the standard electrode for HER, i.e., Pt. MoS_2_ thin films can be engineered to produce active sites for HER. We prepared large area thin films of MoS_2_ from a Mo single source precursor (MoCl_5_) by means of spin coating, followed by post-annealing (sulfurization) with an additional sulfur source in an Ar/H_2_ environment. The obtained films have been characterized by Raman, X-ray diffraction (XRD), UV–vis, and X-ray photoelectron spectroscopy (XPS) before and after post-annealing. The obtained MoS_2_ films are found to be active for HER activity. The HER activity for a 10 nm thick MoS_2_ film is determined at an overvoltage of 290 mV, while for 50 nm films, HER activity is observed at 369 mV at a current density of 10 mA/cm^2^. The HER performance of the thinner films of MoS_2_ is better than that of the thicker films of MoS_2_. XPS results show that the obtained MoS_2_ films have sulfur deficiency (S-vacancies), which is beneficial for HER activity. The Tafel slope extracted from the polarization curve is 80 mV per decade, which is superior to those of single-crystal MoS_2_ and other 2D TMD materials.

## Graphical abstract

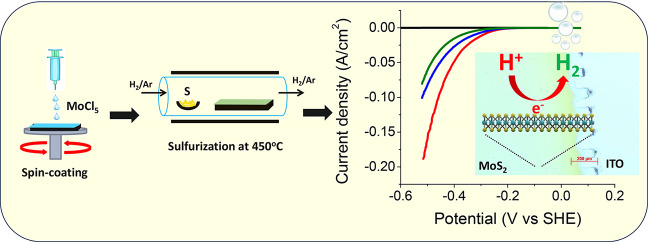

## Introduction

Two-dimensional (2D) transition metal dichalcogenides (TMDs) have gained attention of the material science community in the past decade due to their unique 2D structure, electronic, and catalytic properties that make them suitable for a large range of applications.^1–3^ A suitable catalyst is required for the hydrogen evolution reaction (HER) to lower the overpotential and minimize energy consumption. Among other 2D-TMDs, MoS_2_ is one of the best catalysts for the hydrogen evolution reaction because of its suitable band structure.^4^ It has a bandgap of 1.8 eV for the 2H-phase,^5^ unlike the conducting analogue material, graphene.^6^ Hydrogen can replace currently used fossil fuel in the future due to its high energy density, and it can be obtained from water due to its abundance and harmless byproducts by means of the hydrogen evolution reaction.^7^ The current state-of-the-art catalyst for HER is platinum^8^ due to its exceptional catalytic activity and near-zero overpotential, but due to its low abundance and high cost, it needs to be replaced by some high-earth-abundance and low-cost material such as MoS_2_. Typically, metal oxides are used for oxygen evolution reactions (OER) and energy storage devices,^9–11^ while MoS_2_ is suitable for HER^12,13^ and switching devices^1^ due to its semiconductor-like band structure. MoS_2_ has been proven to be one of the most effective non-precious metal electrocatalysts for HER because of its semiconducting and 2D nature, specifically its edges act as active sites^13,14^ for HER, where the Gibbs free energy for HER adsorption is almost zero.^14^ On the other hand, the basal planes of MoS_2_ are inactive for HER. In order to densify the active sites, different strategies have been reported, such as sputtering MoS_2_ sheets with Ne-ions to produce defects (sulfur vacancies),^15^ doping^16,17^ and phase engineering.^18^ MoS_2_ assists in splitting water into O_2_ and H_2_ electrochemically in one of the two half-cell reactions, where the cathodic part of the reaction is given below

$$2H^{+}+2e\to H_{2}$$

The overall reaction can be expressed as

$$2H_{2}O\to 2H_{2}+O_{2}$$

For mass production, MoS_2_ thin films must be produced on a large scale. Various growth techniques have been reported in order to grow thin films of 2D-TMDs, such as chemical vapor deposition (CVD),^19^ electrochemical deposition,^2^ spray coating,^20^ and dip-coating.^21^ Various deposition methods, such as chemical vapor deposition (CVD), are known to produce high-quality MoS_2_ crystals, but often require complex vacuum processing. In this study, MoS_2_ thin films were fabricated using spin-coating as a more accessible approach using a single-source molybdenum precursor, MoCl_5_. After spin-coating, the precursor undergoes a sulfurization process at 450 °C in an inert atmosphere with an additional sulfur source, converting it into MoS_2_. Spin coating is advantageous for large-area deposition, with the film thickness being primarily determined by the precursor solution concentration and the spin speed. In this work, the spin speed was fixed, while the concentration of the precursor solution was varied to control film thickness (10–50 nm). The resulting MoS_2_ films were evaluated for their hydrogen evolution reaction (HER) activity. HER performance as a function of MoS_2_ film thickness was systematically investigated, with results comparable to those reported for amorphous MoS_2_.^22^ The Tafel slope, extracted from polarization curves, was compared to that of MoS_2_ films produced by other deposition techniques,^2,4,12,20,23–26^ as well as to other transition metal dichalcogenides (TMDs), offering insights into the catalytic efficiency and potential of this fabrication method.

Several studies have also reported the growth of large area MoS_2_ as a catalyst for HER with different growth methods such as hydrothermal growth,^25^ CVD,^13^ and electrodeposition,^2^ and hybridization with conductive support as graphene. Although the CVD processed MoS_2_ monolayer has high crystallinity and exhibits low overpotential (∼180 mV at 10 mA/cm^2^), this approach requires a high-temperature and vacuum-based processor, which limits scalability. Similarly, MoS_2_ films obtained from the hydrothermal process also showed good HER activity, but they often require additional conductive support that reduces film adhesion and uniformity on the substrate. On the other hand, our work uses a facile and scalable spin-coating approach using a single-source precursor, followed by a moderate sulfurization temperature of 450 °C, resulting in the growth of uniform MoS_2_ thin films without the need for a vacuum process. The obtained MoS_2_ films are amorphous in nature, as confirmed by Raman and XRD measurements, but this amorphous structure is beneficial for HER, as it offers denser edge active sites. Our MoS_2_ films show an overpotential of 290 mV at 10 mA/cm^2^ and a Tafel slope of 80 mV/decade, which is comparable to several MoS_2_-based catalysts reported in the literature. This work therefore shows an effective, economical path to grow MoS_2_-based electrocatalysts, making it especially suitable for large-area or industrial-scale hydrogen production applications where material simplicity, processability, and performance are kept balanced.

## Experiment

Molybdenum­(V) pentachloride (MoCl_5_) precursor (as a single source Mo-precursor) was dissolved in 1-methoxy-2-propanol with different concentrations in order to get different film thicknesses after spin coating. The prepared solutions were stirred overnight in order to get uniform closed films. MoCl_5_ is very reactive with organic solvents, e.g., 1-methoxy-2-propanol, resulting in HCl formation.^27^ However, the resulting solution is quite stable in air. After preparation of the solution, a 180 nm ITO/glass substrate was cleaned with acetone and 2-propanol and dried with a nitrogen gun. The substrate was heated at 120 °C for 15 min to evaporate the residual solvents and was afterward treated with UV-ozone to remove organic contamination. The sheet resistance of the bare ITO substrate was measured to be 12 ± 2 Ω/sq, as determined using a four-point probe setup. The as-prepared solution was spin-coated on the 1 × 1 cm^2^ ITO/glass substrate at 3000 rpm for 1 min. A 1 × 1 cm^2^ active area ITO substrate was defined by applying Kapton tape (or vacuum tape) to make the surrounding area of the rectangular ITO-coated glass substrate. MoCl_5_ precursor was then spin-coated on the exposed 1 × 1 cm^2^ area. After spin-coating, the Kapton tape was removed, leaving behind MoS_2_ coated with a well-defined area of 1 × 1 cm^2^. The uncoated area of the ITO substrate was used for contact during the electrochemical study. The obtained films were preannealed at 150 °C for 20 min to evaporate the remaining solvents. The thickness of the obtained films is directly proportional to the concentration of the solution (see Figure 2a). The samples were then postannealed at 450 °C in a 95% Ar 5% H_2_ environment with an additional sulfur source present. For this purpose, samples were placed in a quartz tube, and the tube was inserted into an oven. Pure sulfur (4.5 g) was kept in a separate container in the upstream near the edge of the oven. The air in the quartz tube was replaced by the Ar/H_2_ gas with a high flow rate for 3 min. The temperature of the oven was raised from room temperature to 450 °C at a rate of 5 °C/min. The target temperature was maintained for 120 min. The chemical reaction that occurs during the process is given below.^28^ The complete experiment is illustrated in Figure 1.

**Figure 1:**
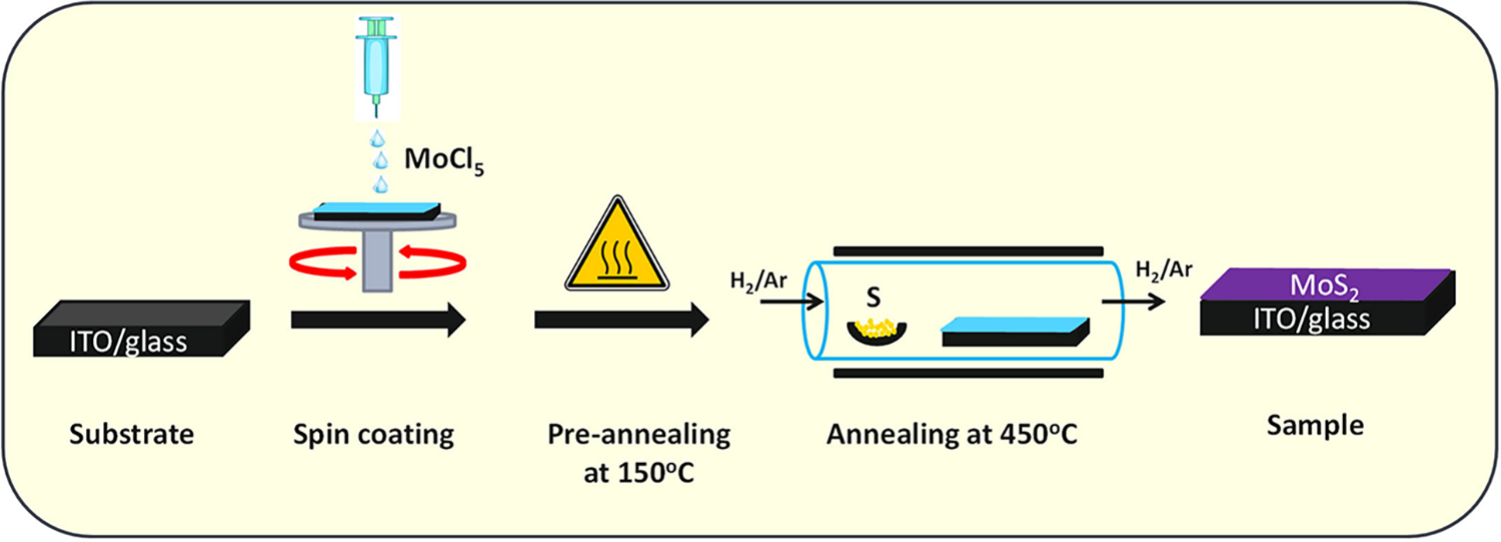
Schematic representation of the experimental process: spin coating of the Mo precursor followed by annealing-produced MoS_2_ thin films.

$${\mathrm{MoCl}}_{5}\overset{\mathrm{ROH}/-\mathrm{OH}}{\to }{\mathrm{MoCl}}_{x}{\mathrm{OR}}_{y}+S_{8}H_{2}\overset{-\mathrm{HCl},-H_{2}O,H_{2}S}{\to }{\mathrm{MoS}}_{2}$$

The obtained films were characterized by different spectroscopic techniques, i.e., Raman, UV–vis, EDS, XRD, and XPS, to investigate the composition of the films and their crystal quality. Scanning electron microscopy (SEM) was done using Zeiss Supra VP55 (Carl Zeiss AG) at an accelerating voltage of 12 kV, with a working distance of 8 mm and aperture size of 60 μm. Elemental analysis was performed by detecting the energy dispersive X-ray (EDX) signal using a silicon drift detector (SDD, Oxford Instruments). To investigate the surface morphology, atomic force microscopy (AFM) (Nanosurf) (tapping mode) was performed. For the Raman measurement, a 514 nm laser was used at 1 mW power. A 50x objective was used to collect the Raman scattered photons. The collected signal was then dispersed with a 2400 g/mm grating, and the signal was detected by a charge-coupled device detector (CCD) cooled by liquid nitrogen (Horiba Jobin-Yvon T64000). The UV–vis measurements of the films were done using a Cary 5000 UV Vis NIR spectrophotometer in the spectral range from 200 to 1100 nm. For UV–vis measurements, the films were deposited on quartz substrates. X-ray diffraction (XRD) measurements were performed using a Bruker AXS Advance D8 equipment set up in grazing incidence mode. The measurements utilized a Cu K_α_ radiation source (λ = 1.5406 Å) with an excitation voltage of 40 kV and a current of 40 mA. This configuration optimizes the detection of diffraction patterns in polycrystalline thin films, providing insights into the structural properties of the MoS_2_ film deposited on a silicon substrate. For X-ray photoelectron spectroscopy (XPS), the layers were deposited on a gold-coated silicon wafer substrate to avoid charging during the XPS measurements. Photoelectrons were excited by Mg *K*_∝_ radiation (*E* = 1253.6 eV) from an Mg/Al X-ray gun (Specs XP-50). The analyzer was operated in fixed analyzer transmission mode with a pass energy of 50 eV. The energetic shift in the binding energy position due to minor charging of the sample was corrected with respect to the C 1s peak. The data evaluation was done by using CASAXPS software. Shirley’s method was used to subtract the background. All of the above characterizations were performed on ∼10 nm MoS_2_ films. After spectroscopic measurements, the samples were used for the hydrogen evolution reaction. For this purpose, 0.5 M sulfuric acid (H_2_SO_4_) was used at pH = 0. A Gamry potentiostat (Gamry Interface 1010E) with three electrodes was used: working electrode (MoS_2_ thin films spin-coated on ITO/glass substrate), counter electrode (a platinum wire), and calomel reference electrode (Hg/HgCl). The HER study was done on samples with different thicknesses. To observe HER activity of the MoS_2_ layers, the overvoltage was scanned from 0.1 to −0.7 V with respect to a standard hydrogen electrode (SHE) with a scan rate of 2 mV per second. To obtain the reaction rate of the HER, Tafel slope analysis was done based on the measured polarization curve.

## Results and Discussion

In Figure 2a, the thickness and the roughness of the spin-coated films against the concentration of the precursor are shown. The film thickness was measured with a Dektak profilometer. The thickness of the films shows a linear correlation with the concentration of the precursor solution. Films down to a few nm were successfully deposited with surface roughness less than 1 nm. The surface roughness of the grown films is found to be independent of the thickness of the films (Figure 2b). The average roughness is lower than 1 nm, which is comparable to the thickness of a monolayer of MoS_2_.^1^ The low roughness of the films also indicates complete coverage of the surface. The films annealed at 450 °C have an amorphous nature, and the crystallinity of the film increases with annealing temperature as reported in our previous work.^28^ 450 °C is the upper limit for annealing because the underlying conducting ITO layer is not stable at higher temperatures. The spin-coated source Mo-precursor was successfully converted to MoS_2_ by post-annealing at 450 °C in a 95% Ar/5%H_2_ environment with an additional sulfur source. The reduction of the Mo-precursor to MoS_2_ during post-annealing is studied by Raman, XRD, UV–vis, and XPS spectroscopy. Hydrogen gas aids in removing organic impurities from the solvent used during the preparation of the precursor solution (prior to spin coating). Additionally, it facilitates the reduction of the molybdenum precursor to MoS_2_ at a lower temperature.^29^ The boiling point of sulfur is 444.6 °C, and the high vapor pressure helps in the diffusion of the sulfur atoms into the film and the reduction of the precursor to MoS_2_. Figure 2c displays an optical microscope image of the annealed sample, with the uniform color contrast indicating consistent film uniformity. Figure 2d–f presents SEM images and EDX analysis of the MoS_2_ film. The EDX data show the presence of molybdenum (highlighted in yellow, Figure 2e) and sulfur (shown in purple, Figure 2f) distributed across the sample. This confirms that the initially spin-coated Mo precursor was fully sulfurized during postannealing. The EDS spectrum is provided in the Supporting Information in figure S2.

**Figure 2:**
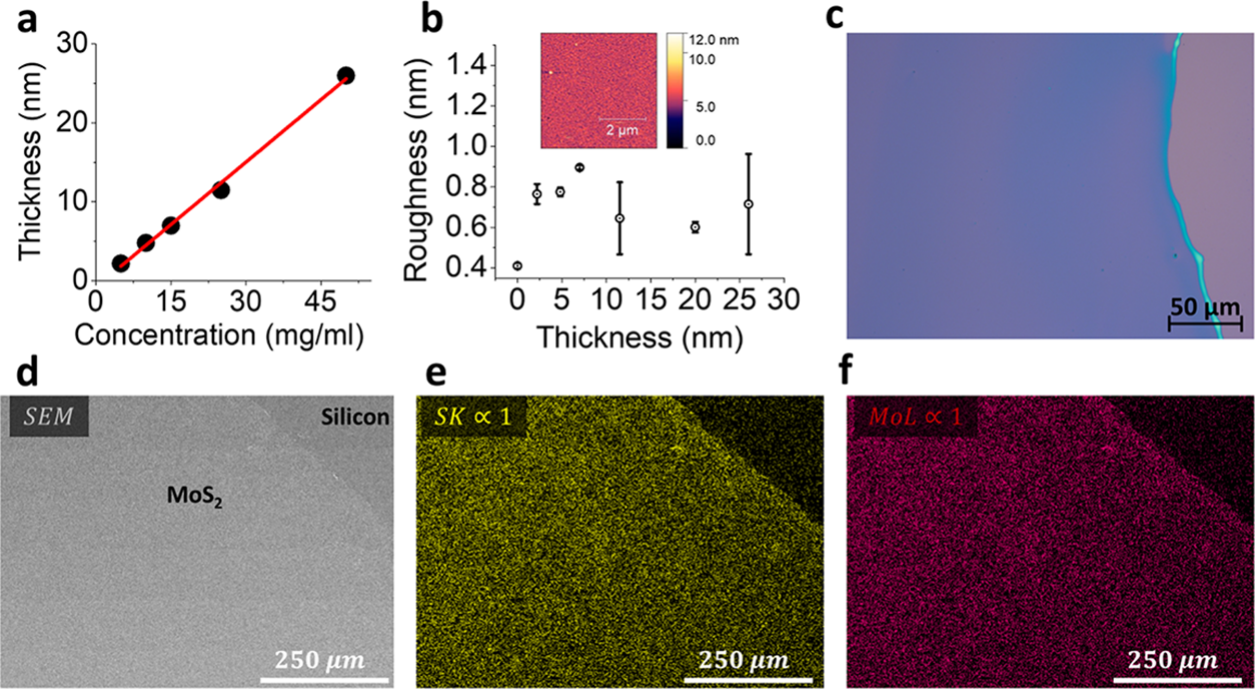
(a) Film thickness as a function of MoS_2_ precursor concentration. (b) Thickness versus surface roughness with an inset showing the AFM image (scale bar: 2 μm) of the MoS_2_ film. (c) Optical microscope image of the MoS_2_ film on silicon wafer with 270 nm SiO_2_. (d) SEM image of the MoS_2_ film on a silicon wafer. (e) EDS mapping showing sulfur distribution in yellow. (f) EDS mapping showing molybdenum distribution in purple.

Raman spectroscopy is a nondestructive way to study the TMDs via their phonon vibrations. In Figure 3a, peaks at 383 and 408 cm^–1^ are visible, which correspond to the Raman active modes of MoS_2_.^30^ The peak at 408 cm^–1^ is related to the A_1g_ vibrational mode, which is an out-of-plane vibration, while the peak at 383 cm^–1^ corresponds to the E_2g_^1^ vibrational mode, which is an in-plane vibration.^30^ According to theoretical prediction, there are four Raman active modes of MoS_2_, of which we observe only two in the measured spectral range.^31^ From literature it is known that the difference between A_1g_ and E_2g_^1^ modes for the monolayer of MoS_2_ is 18 cm^–1^ and for the bulk-MoS_2_ it is 25 cm^–1^.^28,30^ In the measured Raman spectrum (Figure 3), a difference of 25 cm^–1^ is observed between A_1g_ and E_2g_^1^ modes. This implies that the obtained films are bulk-like MoS_2_. The full width at half-maximum (FWHM) of the Raman peak gives us information about the crystal quality. Reported studies^28,32^ show that the FWHM of the A_1g_ Raman mode for a perfect crystal (mechanically exfoliated flakes) is 1.97 cm^–1^, in agreement with our own measurement. FWHM goes up as the quality of the crystal decreases.^32^ The FWHM of A_1g_ mode in Figure 3a is 8.1 cm^–1^, which means that the crystallinity of the obtained MoS_2_ film is low or the material tends toward an amorphous state. XRD measurements were performed on MoS_2_ films deposited on a silicon wafer. In Figure 3b, XRD spectra of MoS_2_ annealed at 750, 600 and 450 °C are shown. In the spectrum of MoS_2_ annealed at 750 °C, a prominent diffraction peak corresponding to the (002) plane of MoS_2_ is observed at 2θ = 14.4°, indicating a well-formed layered structure in the 2H phase.^28^ Additionally, faint higher-order reflections, such as the (004) peak, appear at 2θ = 26.1°. Although these higher-order peaks are weak, their presence suggests partial crystallinity, and further annealing at higher temperatures could improve the overall crystalline quality of the film. The sample annealed at 600 °C displays a broader and less intense (002) peak, indicating the presence of MoS_2_ with reduced crystallinity. This broadening suggests smaller crystallite sizes or a higher degree of structural disorder within the film. In contrast, the MoS_2_ peak is absent in the sample annealed at 450 °C, indicating that the film remains amorphous at this temperature. This observation is supported by Raman spectroscopy, which only shows broad peaks corresponding to MoS_2_ vibrational modes, further confirming the lack of long-range crystalline structure. These results indicate that the structural quality and crystalline orientation of MoS_2_ improve with increasing annealing temperature. At higher temperatures, sufficient thermal energy overcomes surface energy barriers, promoting grain growth and improving the ordering of MoS_2_ layers. The observed (002) and (004) peaks correspond to an interlayer spacing (*d*) of approximately 6.15 Å, consistent with values reported for 2H-MoS_2_ in the literature.^33,34^

**Figure 3:**
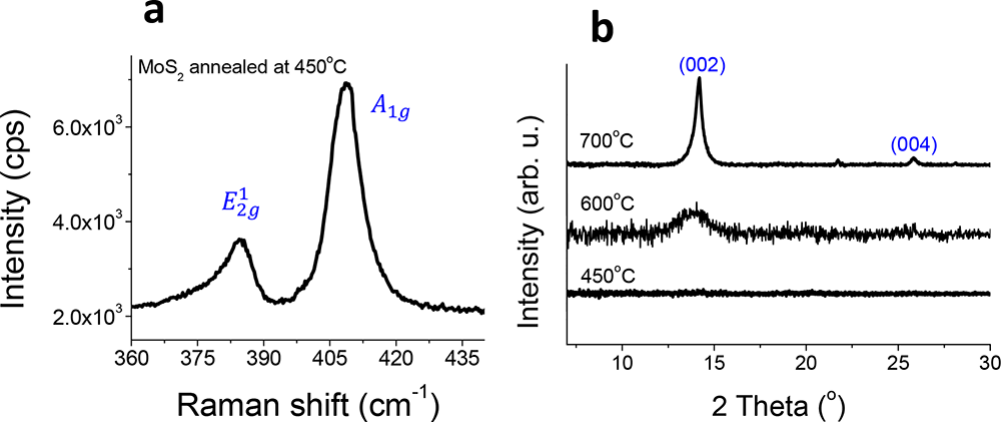
(a) Raman spectrum of the MoS_2_ film annealed at 450 °C. (b) XRD spectra of MoS_2_ annealed at different temperatures.

In Figure 4, the UV–vis spectra of the spin-coated film before and after annealing are shown. UV–vis absorption measurements were done for the spin-coated and annealed samples to further verify the conversion of the spin-coated Mo precursor to MoS_2_. The spin-coated film before annealing exhibited no clear absorption peak, but absorption was slightly increased beyond 400 nm. The lack of characteristic peaks on MoS_2_ before annealing indicates the absence of any MoS_2_ content in the as-deposited film. The spectrum of the annealed sample exhibits clear characteristic broad peaks of MoS_2_ at 612 and 674 nm, which indicates the conversion of the as-deposited Mo precursor to MoS_2_ after postannealing. The peaks at 612 and 674 nm correspond to the A and B excitons of MoS_2,_ respectively, which originate from a direct transition at the K point of the Brillouin zone.^35^ In addition, one broad peak is observed around 400–450 nm, which corresponds to the C and D excitons, corresponding to further interband transitions.^36^ The broadness of the peaks indicates that MoS_2_ in the film has low crystal quality or is in the amorphous form.^28^

**Figure 4:**
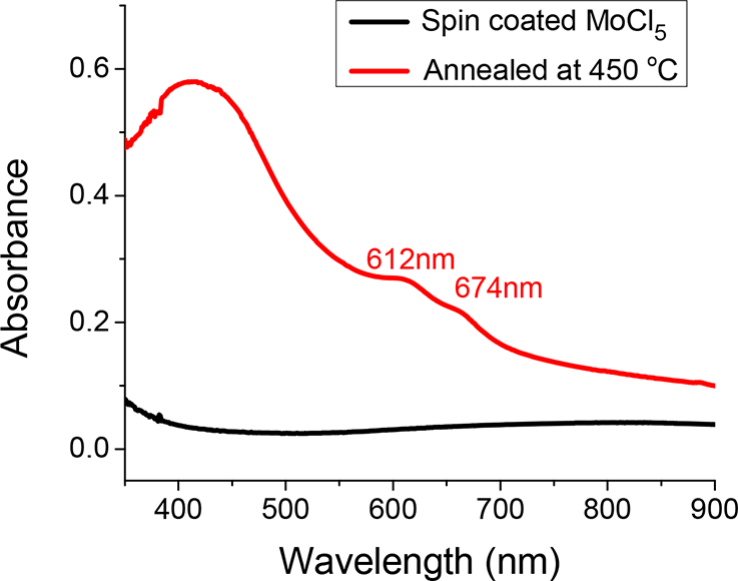
UV–vis spectra of the MoS_2_ film annealed at 450 °C (red) compared to those of the spin-coated MoS_2_ precursor (black).

The films were further characterized by XPS to study the chemical state and composition of the spin-coated films before and after their conversion to MoS_2_. In Figure 5 X-ray photoelectron spectra and corresponding fits for the Mo 3d_3/2_ and 3d_5/2_ doublet of the sample after spin-coating the Mo-precursor and after annealing the sample (Figure 5a), and S 2p_1/2_ and 2p_3/2_ doublet after sample annealing (Figure 5b) are shown. An additional S_2s_ peak appears in the spectrum after annealing (lower panel of Figure 5a). The spectrum of the just spin-coated Mo-precursor mainly consists of a doublet with binding energies of 235.5 and 232.3 eV corresponding to the Mo 3d_3/2_ and 3d_5/2_, respectively, which is attributed to MoO_3_.^37^ In the lower panel of Figure 5a, the spectrum of the annealed film at 450 °C with an additional sulfur source in an inert environment is shown. The major peaks are now located at 232.4 and 229.2 eV, corresponding to Mo 3d_3/2_ and 3d_5/2_. The shift of both 3d states of Mo with respect to the MoO_3_ counterparts is 3.1 eV. The new peak position fell in the range typical for MoS_2_.^32,38^ Furthermore, an additional peak at 226 eV appeared after annealing, which corresponds to the S_2s_ state. The ratio between the Mo_3d_ peak and S_2s_ is found to be 1:1.89, which also confirms the formation of MoS_2_ after annealing, as verified by UV–vis and Raman spectroscopy. The atomic ratio between Mo and S indicates that the sulfur concentration is slightly lower in the MoS_2_ obtained films, in comparison to an ideal MoS_2_ ratio (1:2). The deficiency of sulfur gives rise to point defects in the MoS_2_ crystal, such as S-vacancies.^39^ At the S-vacancy sites, the access of Mo atoms introduces a gap state that increases the efficiency for HER.^19,39^ Raman spectra (Figure S3) and XPS spectra (Figure S4) of spin-coated MoS_2_ films measured before and after electrochemical study are included in the Supporting Information. These results indicate that the MoS_2_ film grown by such a simple method is chemically and structurally stable during HER measurements without using any additional support or binder.

**Figure 5:**
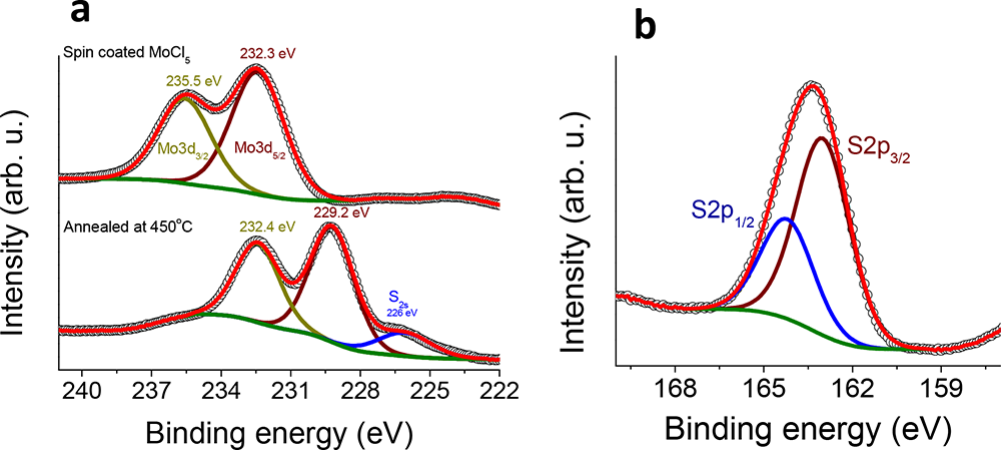
XPS Mo_3d_ spectra with fitted data: (a) Mo_3d_ peak for the spin-coated MoS_2_ precursor (upper panel) and the Mo_3d_ peak along with the S_2s_ peak for the annealed MoS_2_ film (lower panel). (b) S_2p_ peak for MoS_2_ annealed at 450 °C.

To validate the HER activity of the synthesized MoS_2_ films, the films were deposited onto an electrochemically inactive substrate for HER, specifically a 180 nm ITO/glass substrate. Initially, the bare ITO/glass substrate was tested against a standard hydrogen electrode (SHE) to establish its HER performance. The polarization curve for bare ITO/glass (Figure 6a) exhibited a negligible increase in current density, indicating that the ITO substrate is indeed inactive for HER. However, all MoS_2_ films of varying thicknesses deposited on ITO/glass substrates showed clear HER activity (Figure 6a) at a current density of 10 mA/cm^2^, the overpotentials were 290 mV for the 10 nm MoS_2_ film, 323 mV for the 25 nm film, and 369 mV for the 50 nm film. A slight increase in overpotential with film thickness was observed, which can be attributed to the higher series resistance in thicker films, as confirmed by impedance measurements, as can be seen in Figure 6c. The HER in acidic media normally follows the Volmer–Heyrovsky or Volmer–Tafel mechanism.^40^ The mechanism initiates with the adsorption of the hydrogen atom on the MoS_2_ catalyst surface.

$$H_{3}O^{+}+e^{-}\to H^{*}+H_{2}O\;(\mathrm{Volmer}\;\mathrm{step})$$

**Figure 6:**
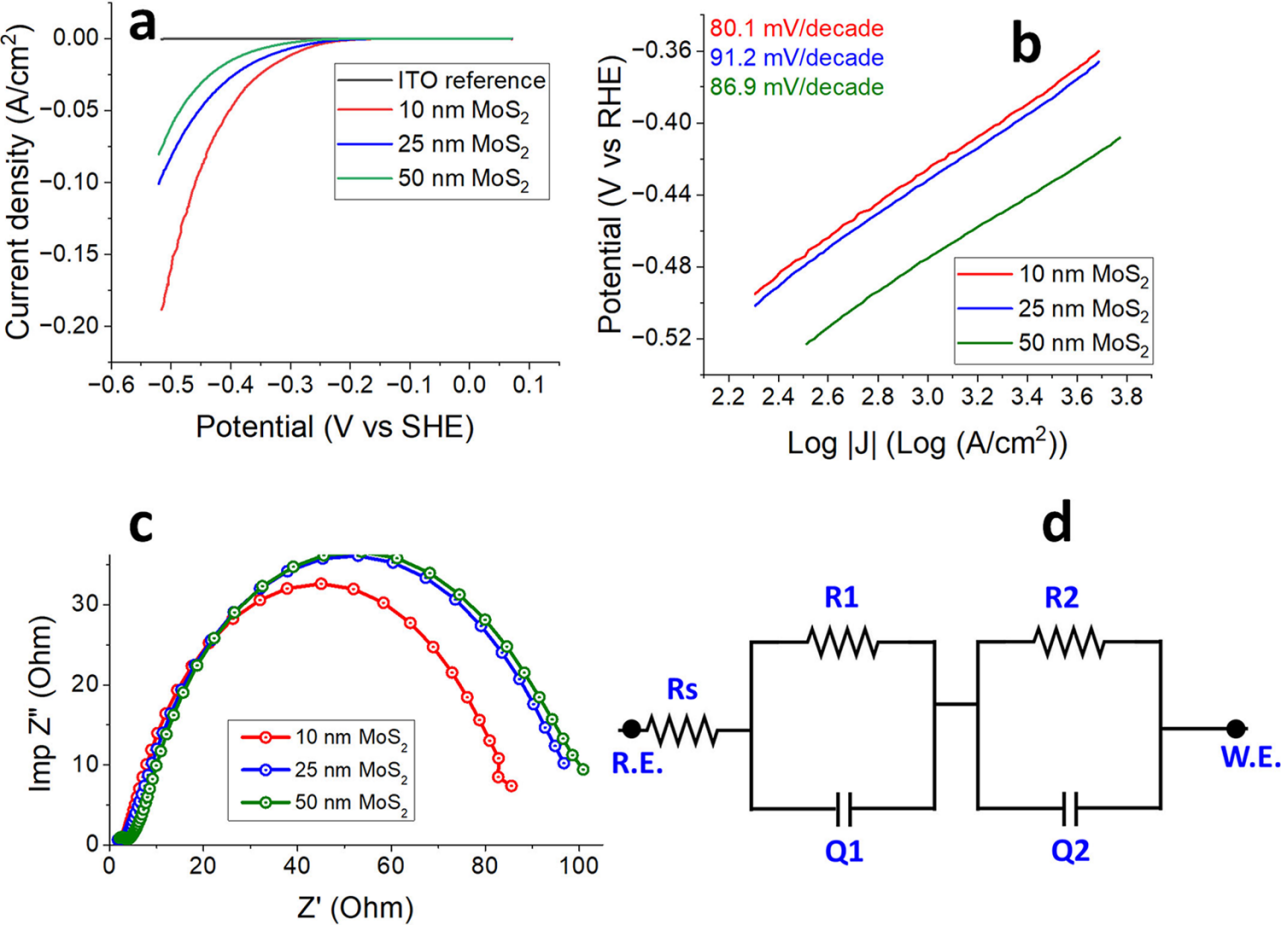
(a) HER polarization curves for MoS_2_ films of varying thicknesses. (b) Tafel slope extracted from Figure 6a for MoS_2_ films of different thicknesses. (c) EIS impedance measurements for MoS_2_ films of varying thicknesses. (d) Equivalent circuit model used for fitting the EIS data of spin-coated MoS_2_ thin film.

In the second step, hydrogen gas is formed with reaction between hydrogen radical (H*) and proton

$$H^{*}+H_{3}O^{+}+e^{-}\to H_{2}↑+H_{2}O\;(\mathrm{Heyrovsky}\;\mathrm{step})$$

Or in the second step, chemical desorption happens

$$H^{*}+H^{*}\to H_{2}↑\;(\mathrm{Tafel}\;\mathrm{step})$$

MoS_2_ exists in two different crystal phases: the 2H-phase, which is semiconducting in nature, and the 1T-phase, which is metallic in nature.^41^ The XRD measurements suggest that the MoS_2_ films obtained in this work are in the 2H-phase. It is known from the literature^40,42^ that basal planes are inactive for HER, while edge sites are mainly active for HER. In such a case, Mo atoms are coordinately unsaturated and can adsorb hydrogen radicals. These Mo edge atoms play an important role in the hydrogen evolution reaction by acting as the main redox-active sites. Additionally, M^4+^ sites are mainly responsible for redox activity in MoS_2_; on the other hand, sulfur atoms provide an electronic environment. The electron transfer during HER is thought to be related to the molybdenum d-orbital, which facilitates adsorption of hydrogen radicals and the reduction step. The XPS results showed that MoS_2_ obtained via the spin-coating process exhibits sulfur deficiency, which likely corresponds to a higher concentration of exposed Mo-edge sites. This chemical characterization indicates a higher density of HER active sites, contributing to good catalytic performance.

To assess the reaction kinetics of the hydrogen evolution process, the Tafel slopes were derived from the polarization curves for the 10, 25, and 50 nm MoS_2_ films (Figure 6b). The analysis, performed over an overpotential range of −0.36 to −0.52 V, yielded Tafel slopes of 80.1, 91.2, and 86.9 mV/decade for the 10, 25, and 50 nm MoS_2_ films, respectively. The Tafel slope is inversely related to the HER reaction rate, meaning a lower Tafel slope indicates a more efficient HER process with a smaller increase in overpotential required for a 10-fold increase in reaction rate. Comparatively, single-crystal MoS_2_ has a reported Tafel slope of 61–74 mV/decade,^22^ indicating better performance, while analogous 2D transition metal dichalcogenides, such as WS_2_, show a poorer Tafel slope of approximately 138 mV/decade.^43,44^

Figure 6c illustrates electrochemical impedance spectroscopy (EIS) measurements in the form of Nyquist plots (*Re*(*Z*) vs *Im*(*Z*)) for MoS_2_ films of different thicknesses. The Nyquist plots show a clear correlation with HER activity, where a smaller semicircle corresponds to better HER performance. The impedance values for the 10, 25, and 50 nm MoS_2_ films were found to be 85, 96, and 102 Ohms, respectively, further supporting the trend that thinner films exhibit lower series resistance and superior HER activity. This suggests enhanced interfacial charge transfer kinetics in the 10 nm MoS_2_ electrode. The corresponding equivalent circuit model used for fitting the Nyquist plots is depicted in Figure 6d. The impedance measurements also align with general trends seen in the literature, where smaller Nyquist plot semicircles correlate with lower charge-transfer resistance and better HER activity. The results obtained from electrochemical impedance spectroscopy are modeled using an equivalent circuit as shown in Figure 6d. The model has solution resistance (*R*_s_) in series with two *R*–*Q* elements in series, showing an electrochemical process happening at the working electrode surface. The first circuit loop with *R*1 and *Q*1 represents the charge transfer resistance and nonideal double layer capacitance at the MoS_2_ film and electrolyte interface. The second circuit loop with *R*2 and *Q*2 is attributed to additional resistance and capacitance originating from internal interfaces, i.e., grain boundaries and MoS_2_ film/ITO substrate interface. Constant phase elements (*Q*1 and *Q*2) are used instead of an ideal capacitor in the model circuit due to the inhomogeneity and amorphous nature of our films, which is verified by Raman and XRD measurements.

To highlight the effectiveness of our spin-coated MoS_2_ thin film as a catalyst for hydrogen production, we prepared a table above that compares the HER performance of our results with the reported catalysts in similar acidic media. Ten nm MoS_2_ film produced by spin coating in our study shows overpotential value 290 mV at 10 mA/cm^2^ and a Tafel slope of 80.1 mV/decade, while these values are not the best among the reported studies, but they are comparable to or better than films produced by some methods discussed below and shown in Table 1.

**Table 1:** Comparison of HER Performance with Reported Catalysts in Acidic Media

catalyst material and growth method	overpotential @ 10 mA/cm^2^ (mV)	Tafel slope (mV/dec)	ref
this work: spin-coated MoS_2_	290	80.1	
platinum state-of-the-art catalyst	28	28	^45^
MoS_2_ atomic layer deposition	266	96	^12^
MoS_2_ spray-coated	500	not mentioned	^20^
MoS_2_/graphene solvothermal reaction	150	41	^26^
MoS_2_ hydrothermal	210–255	50–75	^25^
MoS_2_ chemical exfoliation	250	75–85	^24^
MoS_2_ CVD	210–300	53–110	^23^

For instance, MoS_2_ (2H-phase) thin film grown by atomic layer deposition exhibits an overpotential of 266 mV at 10 mA/cm^2^ and a Tafel slope of 96 mV/decade,^12^ although ALD-grown MoS_2_ films show better HER activity but also require a vacuum process and longer growth time. MoS_2_ monolayer produced by chemical vapor deposition (CVD) also shows good overpotential values of 210–300 mV at 10 mA/cm^2^, and a Tafel slope of 53–110 mV/decade, again this process also requires higher process temperature (>850 °C), multisteps, and is challenging to scale. Similarly, MoS_2_/RGO composite synthesized by the solvothermal approach showed excellent overpotential values of 150 mV at 10 mA/cm^2^ and a Tafel slope of 41 mV/decade,^26^ but this system relies on a conductive support like graphene, making this process complicated.

MoS_2_ films produced by spray-coating exhibit overpotential values of 500 mV at 10 mA/cm^2^.^20^ Meanwhile, hydrothermally produced MoS_2_/MoO_3_ and MoS_2_ (1T-phase) 2D-sheets exfoliated chemically show good HER activity (overpotential of 210–250 mV at 10 mA/cm^2^, Tafel slope of 50–75 mV/decade) due to higher edge sites.^24,25^

Compared to the above literature, our work offers a unique balance between simplicity, scalability, and performance.

## Conclusions

MoS_2_ thin films were successfully deposited using a spin-coating technique with a single Mo-based precursor, followed by postannealing at 450 °C in the presence of an additional sulfur source within an Ar/H_2_ atmosphere. The films exhibited a surface roughness of less than 1 nm, indicating an excellent uniformity and complete surface coverage. EDX analysis confirmed the uniform distribution of MoS_2_ throughout the sample. The films were characterized by Raman spectroscopy, UV–vis, and XPS, both before and after annealing. The resulting MoS_2_ films demonstrated catalytic activity for the hydrogen evolution reaction (HER), with a low Tafel slope of 80 mV/decade extracted from the polarization curve, signifying efficient HER kinetics. Given its low cost and high abundance, MoS_2_ is an appealing alternative to expensive noble metal catalysts for the HER. Spin-coating, being a scalable deposition method, facilitates the production of MoS_2_ thin films over large areas. The approach presented for fabricating HER cathodes represents a promising avenue for producing hydrogen from water as a sustainable fuel source in the future.

## Supplementary Material



## Notes

Optical microscope image of a 10 nm MoS_2_ film on the ITO substrate (Figure S1); energy-dispersive X-ray spectroscopy (EDS) spectrum of a 10 nm MoS_2_ film on a silicon wafer (Figure S2); Raman spectra of MoS_2_ films before and after electrochemical study (Figure S3); and X-ray photoelectron spectroscopy (XPS) spectra of MoS_2_ films before and after electrochemical study, together with 50 nm freshly sputter-coated gold film (Figure S4) (PDF)

## □

Karlsruhe Institute of Technology (KIT), Institute for Applied Materials – Ceramic Materials and Technologies, Karlsruhe 76131, Germany
