# Supplementary material for: Facile Spin-Coated MoS2 Thin Films from a Single-Source Precursor for HER Activity
Source: ACS Appl Energy Mater. 2025 Jun 27;8(13):9497–505. doi: 10.1021/acsaem.5c00619 (PMC12264967; doi:10.1021/acsaem.5c00619)
Supplement: Supplementary file 1 [file ae5c00619_si_001.pdf]

Supporting information

Facile Spin-Coated MoS_2 Thin Films from a Single-Source Precursor for HER Activity

Talha Nisar*[1,2], Muhammad Adeel Asghar[3], Abu Nasar Siddique[4], Ali Haider[3], Kaline Pagnan Furlan[2], Veit Wagner*[1]
1. School of Science, Constructor University, Campus Ring 1, 28759 Bremen, Germany
2. Hamburg University of Technology (TUHH), Institute of Advanced Ceramics, Integrated Materials Systems Group, Denickestraße 15, 21073 Hamburg, Germany
3. Department of Chemistry, Quaid-i-Azam University, Islamabad 45320, Pakistan
4. Institute of Biotechnology and Microbiology, Bacha Khan University Charsadda, Pakistan

Key words: MoS_2, hydrogen evolution reaction, solution-based deposition, 2D-material, transition metal dichalcogenide (TMD), electrocatalyst

Email: talha.nisar@live.com and vwagner@constructor.university

Optical microscope image

Figure S1 shows an optical microscope image of the spin coated 10 nm MoS_2 thin film on ITO substrate. The uniform color contrast through the film in the image shows the uniformity of the spin coated film. The irregular edges on the right side of the film are because of slight penetration of the precursor solution beneath the Kapton tape due to centrifugal force during the spin coating process. The tape was removed after spin coating.

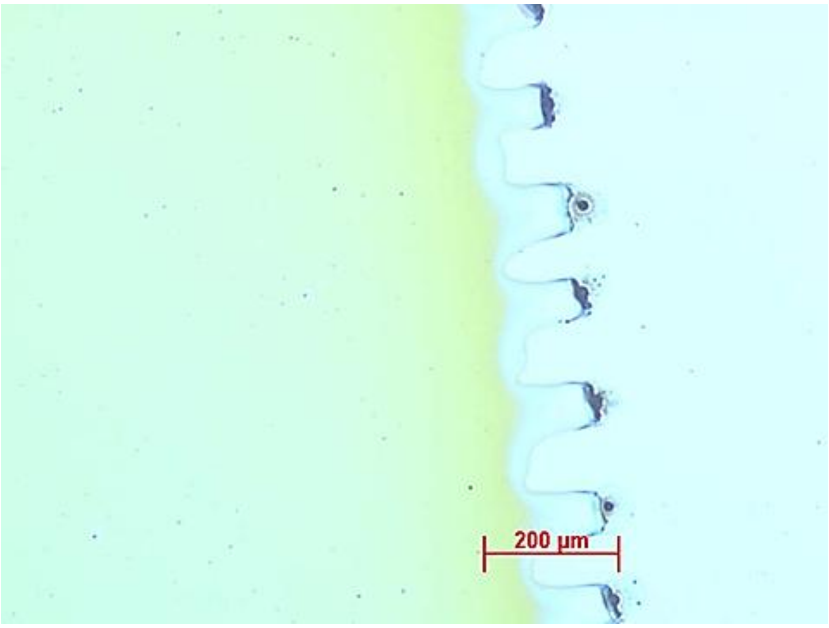

Figure S1. Optical microscope image of spin-coated MoS_2 on ITO/glass substrate (scale bar: 200μm).

Energy Dispersive x-ray spectroscopy

Figure S2 shows the EDS spectrum of the spin-coated MoS_2 thin film on silicon wafer. The spectrum exhibits a broad peak around 2.3 keV corresponding to Mo Lα and S Kα emission line [2], together with strong peak at 1.74 keV which corresponds to Si Kα [3]. The detection of Molybdenum and sulfur peaks in the spectrum confirms the presence of these elements in the obtained film, which is consistent with Raman, UV-Vis, XRD and XPS results. The strong silicon peak is expected, given the thickness of the MoS_2 layer (~10 nm) and depth sensitivity of this process, in which high energy electrons are penetrated deep (~ 5 μm) into the sample. The peak at 0.53 keV corresponds to O Kα [3], which primarily comes from the native SiO_2 layer on the silicon wafer. The peak at 0.23 keV corresponds to C Kα [3], which is attributed to the surface adsorbed hydrocarbons or minor surface contaminations due to the exposure to ambient air during transfer.

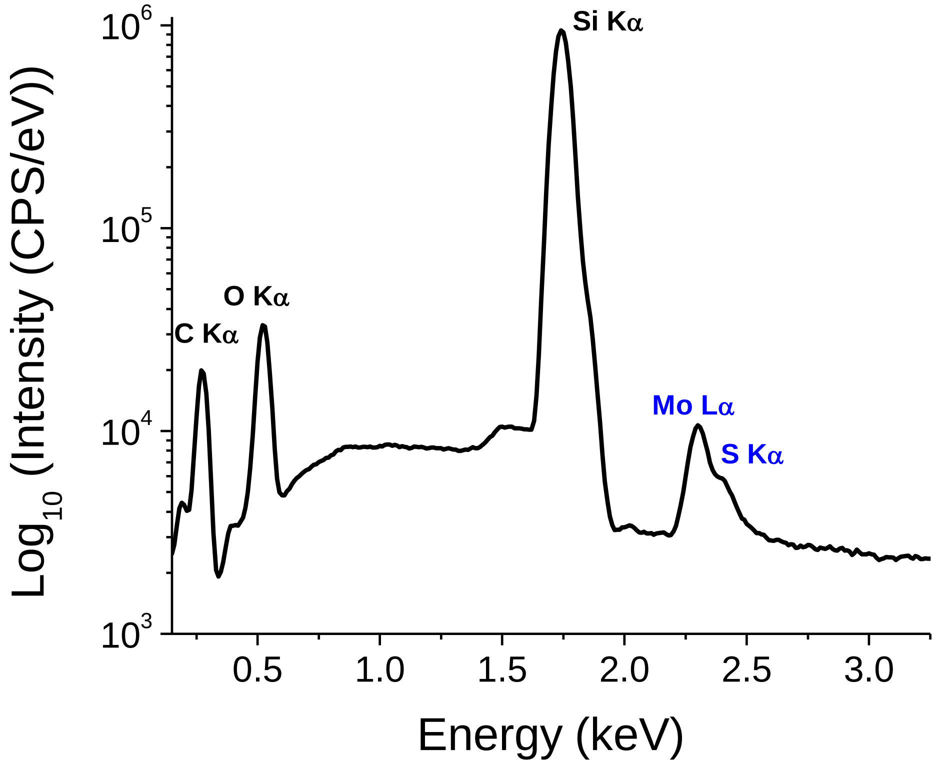

Figure S2. Energy Dispersive x-ray spectroscopy spectrum of spin-coated MoS_2 on silicon wafer.

Raman spectroscopy

Figure S3 shows the Raman spectra of the spin-coated MoS_2 thin films before and after the hydrogen evolution reaction measurements. In both spectra, Raman active modes of MoS_2 are visible: E_{2g}^1 mode at 383 cm^{-1} which corresponds to the in-plane vibrations of Mo and S atoms and A_{1g} mode at 408 cm^{-1} which corresponds to the out of plane vibrations of S atoms. The presence of Raman characteristic peaks in both spectra indicates that the MoS_2 film remained unchanged during the electrochemical study. Additionally, full width at the haft maximum (FWHM) of the A_{1g} peak for both spectra remain same value of 8.1 cm_{-1} which indicates that the crystallinity of the MoS_2 films does not change during the electrochemical study.

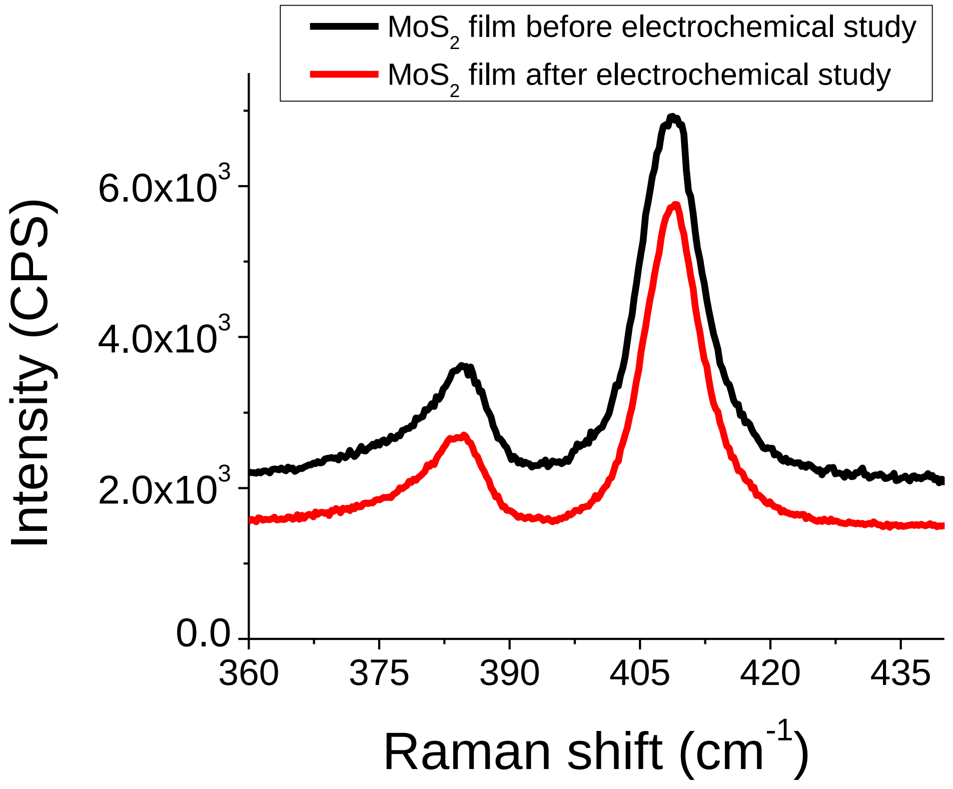

Figure S3. Raman spectra of spin coated MoS_2 films before and after electrochemical study.

X-ray photoelectron spectroscopy (XPS)

Figure S4 shows the XPS overview spectra of spin coated MoS_2 films before and after electrochemical study, together with spectrum of freshly sputter coated gold film on silicon wafer. The spectra of MoS_2 before and after electrochemical study exhibits typical photoelectron peaks related to molybdenum and sulfur, i.e. Mo_{3d}, Mo_{3p}, Mo_{3s}, S_{2s} and S_{2p}, showing that the composition of the films remained unchanged during HER measurements.

In the pre-electrochemical study spectrum, minor Si_{2s} and Si_{2p} peaks are also visible, which originates from the silicon wafer. The O_{1s} peak is attributed to the surface adsorbed contaminations and native oxide layer of the silicon wafer. In the post-electrochemical study spectrum weak Sn and In peaks are visible which is coming from the ITO substrates as the HER measurements were performed on the MoS_2 coated on ITO substrates. O_{1s} peak with lower intensity is also visible which is again attributed to the surface contamination. C_{1s} peak is also visible in both spectra and is attributed to common surface adsorbed contaminations.

To further validate our hypothesis, 50 nm gold was sputter coated on a silicon wafer and was immediately introduced into the XPS chamber. In the spectrum of gold layer, O_{1s} and C_{1s} peaks are visible, even in this freshly prepare and chemically inert system.

Hence, both Raman and XPS measurements clearly shows that structure and composition of the spin coated MoS_2 remain unchanged during HER measurements. This demonstrates the electrochemical stability of the spin coated MoS_2 film in acidic environment. Our results are consistent with previously reported work, where MoS_2 stability was tested during electrochemical study by monitoring the overpotential for over 200 hours of continuous operation and MoS_2 films was proven to be stable on long term, with only 11% loss in catalytic activity [1].

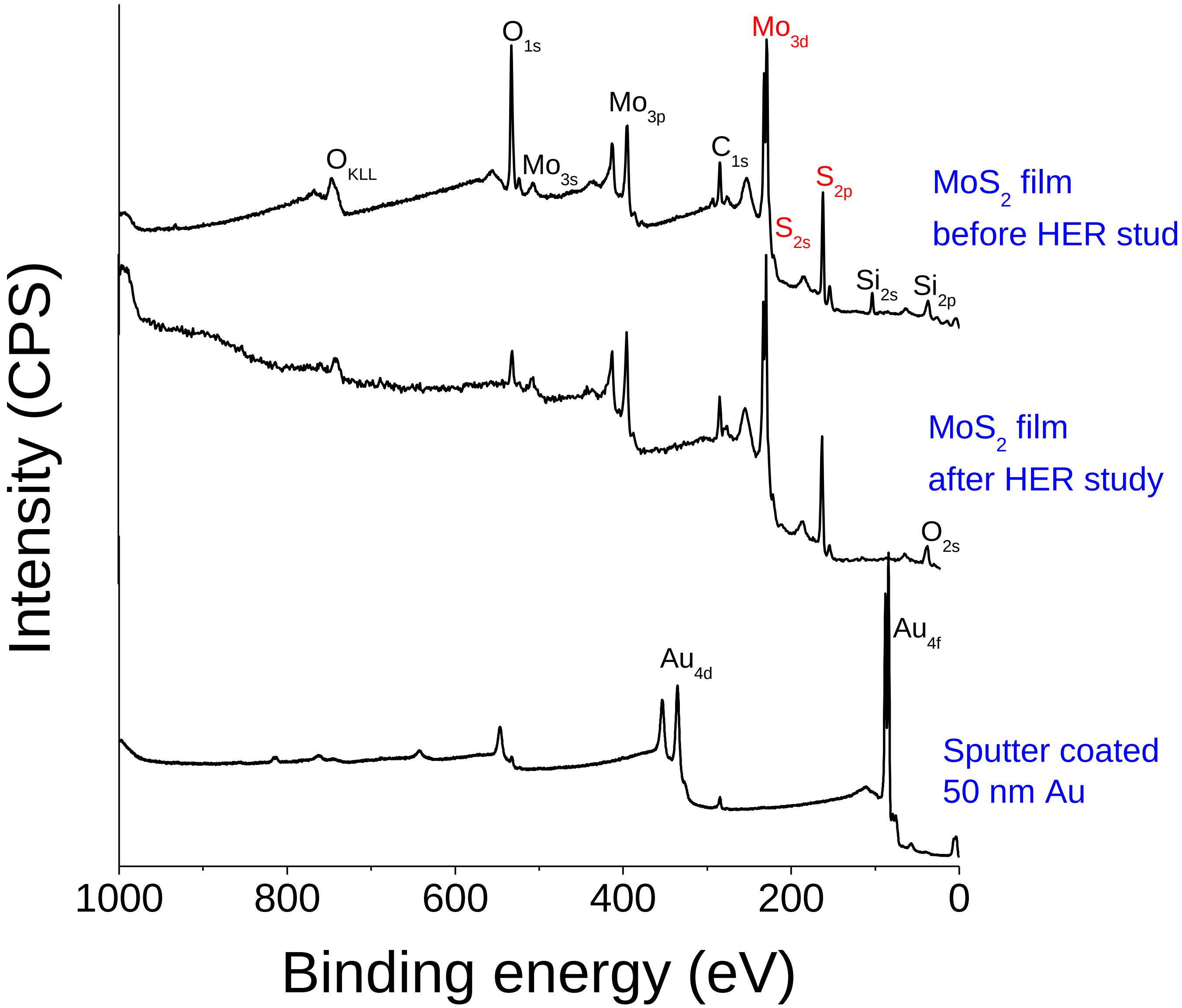

Figure S4. XPS overview of spin coated MoS_2 before and after electrochemical study and sputter coated gold as reference.

References

[1] Vedhanarayanan B, Shi J, Lin J Y, Yun S and Lin T W 2021 Enhanced activity and stability of MoS2 through enriching 1T-phase by covalent functionalization for energy conversion applications *Chemical Engineering Journal* **403** 126318

[2] Govindasamy M, Chen S M, Mani V, Akilarasan M, Kogularasu S and Subramani B 2017 Nanocomposites composed of layered molybdenum disulfide and graphene for highly sensitive amperometric determination of methyl parathion *Microchimica Acta* **184** 725–33

[3] Bahari A, Ghovati M and Hashemi A 2019 Studying of SiO2/capron nanocomposite as a gate dielectric film for improved threshold voltage *Appl Phys A Mater Sci Process* **125** 125–7
